# Alteration of the Respiratory Microbiome in Hospitalized Patients with Asthma–COPD Overlap during and after an Exacerbation

**DOI:** 10.3390/jcm12062118

**Published:** 2023-03-08

**Authors:** Ahmad R. Alsayed, Anas Abed, Yazun Bashir Jarrar, Farhan Alshammari, Bushra Alshammari, Iman A. Basheti, Malek Zihlif

**Affiliations:** 1 Department of Clinical Pharmacy and Therapeutics, Faculty of Pharmacy, Applied Science Private University, Amman 11931-166, Jordan; 2 Pharmacological and Diagnostic Research Centre, Faculty of Pharmacy, Al-Ahliyya Amman University, Amman 11931-166, Jordan; 3 Department of Basic Medical Sciences, Faculty of Medicine, Al-Balqa Applied University, Al-Salt 19117, Jordan; 4 Department of Pharmaceutics, College of Pharmacy, University of Hail, Hail 2440, Saudi Arabia; 5 Department of Medical Surgical Nursing, College of Nursing, University of Hail, Hail 2440, Saudi Arabia; 6 Faculty of Pharmacy, Sydney University, Sydney, NSW 2006, Australia; 7 Department of Pharmacology, School of Medicine, The University of Jordan, Amman 11942, Jordan

**Keywords:** asthma, COPD, asthma–COPD overlap, respiratory, microbiome, personalized medicine

## Abstract

The immediate aim of this study was to comparatively examine the bacterial respiratory microbiome of patients in a stable state and during an exacerbation of asthma–COPD (chronic obstructive pulmonary disease) overlap (ACO). This prospective observational study took place in Jordan between 1 September 2021 and 30 April 2022. Sputum samples from patients with recognized ACO were acquired within 48 h of the exacerbation onset and again at 3 weeks following the exacerbation. The next-generation sequencing Illumina MiSeq was employed and uncovered significantly high bacterial diversity in the sputa. The results showed a significant decrease in the taxonomic richness in the sputum samples collected during the exacerbation episodes compared with those collected from patients in a stable state (*p* = 0.008), with an increase in the taxonomic evenness (*p* < 0.005). This change in the composition of the airway bacterial community suggests that the replacement of a significant portion of the airway microbiome with certain microorganisms may play a role in the decrease in microbial diversity observed during an ACO exacerbation. Greater knowledge of this link could allow for a more focused administration of antibiotics, especially during exacerbations, improving clinical efficacy and patient outcomes.

## 1. Introduction

In clinical practice, patients with both asthma and chronic obstructive pulmonary disease (COPD) are frequently encountered. This observation led to the development of the term “asthma–COPD overlap” (ACO), which describes a group of clinical characteristics rather than a single entity [1,2].

There is still a lack of consensus over a single definition of ACO recognized by all. Age greater than 40 years, persistent airway obstruction, and a history of asthma or evidence of partial bronchodilator reversibility are essential components of most of the criteria presented [3].

The 2020 Global Initiative for COPD (GOLD) update abandoned the phrase “asthma COPD overlap”, stating that asthma and COPD are separate illnesses that may share traits such as eosinophilia or reversibility [4].

The estimated incidence of ACO is 2 to 3%, whereas the prevalence of asthma and COPD is 5 to 17% and 2 to 12%, respectively. Without a consensus on the precise definition of ACO, it is impossible to determine the exact disease burden [3,5].

COPD is a major cause of hospitalization [6]. Deteriorations in respiratory symptoms beyond the usual daily symptoms, termed acute exacerbations of COPD, leads to faster lung function decline [7], high morbidity [6], and mortality [8].

It was suggested that healthy respiratory microbiota is distinct from that present in respiratory disorders [9,10]. Even when the patients are characterized by a clinically stable condition, they have lower airways displaying bacterial colonization [9,10,11]. Nevertheless, since the majority of research projects in this domain have been cross-sectional [10], it has not been possible to identify the nature of the relationship between the assessed outcomes and bacterial colonization. It is crucial to determine whether microorganisms are associated with exacerbations. According to the studies, approximately 50–75% of COPD exacerbations are associated with respiratory infections, depending on the culture techniques employed [6,12,13,14].

Additional efforts are needed to better understand asthma and COPD phenotypes and endotypes, as well as to identify biomarkers that can help to distinguish which patients would be most responsive to specific therapies.

Some studies have looked into the changes in the microbiome of COPD and asthmatic patients. However, the microbiome among ACO patients is still unclear [10,15,16]. Profiling of bacterial populations in the airways of ACO patients will enhance the understanding of the relationship between the respiratory microbiome and the development and outcome of the illness. A better knowledge of this link should allow for a more focused use of antibiotics, especially during exacerbations, hence enhancing therapeutic efficacy and the outcome for ACO patients.

In Jordan, ACO is a factor that increases the likelihood of incurring higher healthcare costs. In addition to being associated with older ages, higher respiratory expenditures are associated with greater disease severity [17].

Accordingly, this study hypothesized that exacerbation of ACO is linked to alterations in the content of the respiratory microbiome. To the best of our knowledge, there is a lack of information regarding this association. Therefore, the immediate aim of this research was to examine the bacterial respiratory microbiome in the stable state (ST) and during an exacerbation for ACO patients. Next-generation sequencing (NGS) was used to determine the respiratory microbiome ecology and detect the bacteria that were less frequently associated with culture challenges and that remained untargeted by usual antibiotics. The findings of this study can help us better understand the bacterial profile during ACO exacerbations, which can help us prescribe appropriate antibiotics and probiotics to ACO patients.

## 2. Materials and Methods

### 2.1. Study Design and Clinical Features

This prospective observational study took place over eight months, from 1 September 2021 to 30 April 2022. Based in Jordan, the study group participants were hospitalized ACO sufferers. Ethical approval was obtained from the Islamic hospital ethical committee in Amman, Jordan (IRB: 101/2021/1053). Informed consent was obtained from the patients.

The respiratory consultant in the hospital (Amman, Jordan) diagnosed ACO according to the GINA and GOLD guidelines [4,18]. Some of the clinical characteristics that have been associated with ACO include an age of 40 years or older, respiratory symptoms including exertional dyspnea, persistent partially reversible airflow obstruction (without normalization of obstruction), and a history of atopy and/or allergies [19,20,21]. Other clinical criteria include a smoking history of at least ten pack-years and an asthmatic history established before the age of forty.

In addition to determining the intensity, duration, and frequency of respiratory symptoms (such as dyspnea and productive cough), patients were also questioned about any prior asthma or allergic rhinitis diagnoses or symptoms. This enabled researchers to identify whether the patients’ exercise ability was restricted.

### 2.2. Sputum Samples and Isolation of Nucleic Acid

Sputum samples from patients with recognized ACO were acquired for standard evaluation. When possible, expectorated sputum samples were obtained within 48 h of the exacerbation onset during hospitalization and 3 weeks following the exacerbation as an outpatient at ST. The salivary contents were separated from the sputum samples to minimize oral bacterial contamination. However, recent evidence indicated that salivary contamination of expectorated sputum had no effect on most sputum samples and that observations of high bacterial diversity likely properly reflect the taxa present in the lower airways [10,22]. The participants collected their sputum samples in sterile 30-mL conical tubes and were informed that the sputum is the viscous substance that comes from deep within lungs, not the watery fluid in the mouth. Participants were instructed to spit out saliva and rinse their mouths with tap water before taking a deep breath and coughing vigorously to evacuate mucus from the lungs. The sputum was then expectorated directly into a collection tube. The samples were processed and frozen at −80 °C for further examination. The genomic lab received frozen specimens on dry ice.

**Figure 1:**
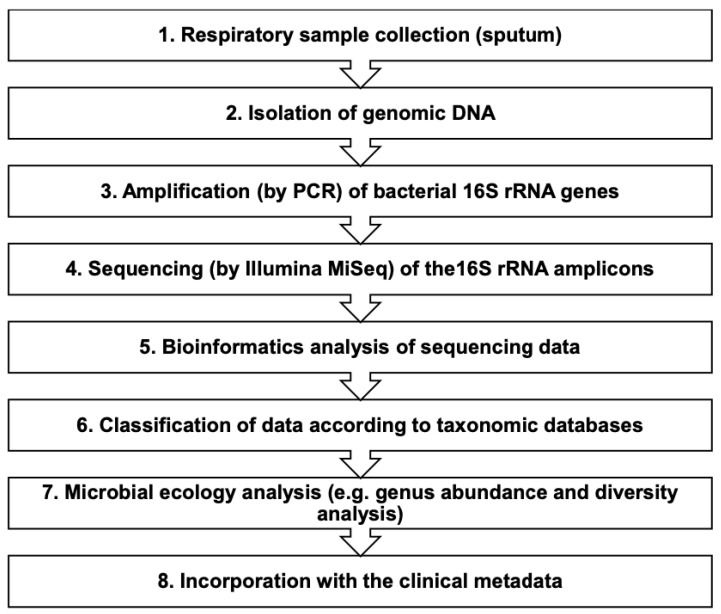
Flow diagram for the microbiome analysis of respiratory specimens (sputa). PCR: polymerase chain reaction; rRNA: ribosomal ribonucleic acid.

The fully automated total nucleic acid extraction BIOBASE Kit was used for gDNA extraction of 200-μL sputum samples using the magnetic beads method (Biobase Biodustry (Shandong) Co., Ltd., Jinan, China). A 1.8-mL sterile phosphate buffered saline solution was added to each sample tube of pre-aliquoted Sputolysin stock to achieve 10% Sputolysin solutions. Following extraction, the eluted 100-μL samples were deeply frozen to be used for library preparation. DNA extracts of the sputum samples were measured for DNA concentration via FLUOstar Omega (BMG Labtech, Germany). Figure 1 represents the flow diagram for the microbiome analysis of the sputum samples from the ACO cohort. In this study, we followed the methods used in previous studies [23,24,25] with some modifications as appropriate.

### 2.3. Library Preparation for the Illumina MiSeq

The library represents a collection of millions of DNA fragments sharing the same known short sequences (adapters) added to the 5′ and 3′ ends. Library preparation is important to ensure the highest quality sequencing data.

The primer pair sequences of the variable V4 region construct a ~300 bp single amplicon. The adapter appended to the primer pair sequences for compatibility with the Illumina MiSeq index and sequencing adapters—the ligation of the amplicon DNA to the adapters—is critical for sequencing as the adapters enable the DNA to hybridize the surface of the sequencing reaction chip. The final length of the template including adapters was ~453 bp.

The following protocol (four PCR steps) represents the procedure for sample preparation for sequencing of the 16S rRNA gene V4 region.

#### 2.3.1. Pre-Amplification of the 16S rRNA Marker Gene Region (PCR 1)

This PCR 1 step was required for the template with a potentially low biomass to pass an adequate tagged amplicon to the final stages related to the indexing amplification. PCR was performed using approximately 200 ng gDNA from every specimen. PCR amplification of the bacterial 16S rRNA gene was constructed by the primers (MWG Eurofins, UK), as presented in the supplementary data. Using the non-modified primers targeting positions 515F and 806R within the V4 region of the 16S rRNA marker gene, a master mix solution (Life Technologies, UK) was prepared, and the amplification was performed using the PCR cycling conditions outlined in the supplementary data. Consequently, an AxyPrep Mag PCR kit was used to clean up the PCR products from PCR 1 (Life Technologies, UK) (supplementary data).

#### 2.3.2. Reverse-Tagging Step (PCR 2)

We performed one PCR cycle using 10 μL of the cleaned PCR 1 product and an equimolar mixture of the reverse frameshift primers (supplementary data). The primers were included in a 0.5-μM working stock. A master mix solution (Life Technologies, UK) was prepared as described in the supplementary data, and the amplification was performed using specific PCR cycling conditions. Consequently, an AxyPrep Mag PCR kit was used to clean up the PCR products from PCR 2 (Life Technologies, UK) (supplementary data).

#### 2.3.3. Forward-Tagging Step (PCR 3)

We performed one PCR cycle using 10 μL of the cleaned PCR 2 product and an equimolar mixture of the forward frameshift primers (Supplementary Data). The primers were included in a 0.5-μM working stock. A master mix solution (Life Technologies, UK) was prepared and the amplification was performed using the PCR cycling conditions (Supplementary Data). Consequently, an AxyPrep Mag PCR Clean-up kit was used to clean up the PCR products from PCR 3 (Life Technologies, UK) (Supplementary Data).

#### 2.3.4. Nextera-Adapter/Indexing Amplification Step (PCR 4)

In this step, we used 15 µL of reverse- and forward-tagged products in a 34-cycle PCR targeting the 16S rRNA V4 region from the third PCR step that had been cleaned. Each reaction had identical forward primers and a reverse primer that served as the sample’s barcode. Both primers were diluted to a 5-µM stock. They were added to every reaction. A master mix solution was used as described in the Supplementary Data. The forward primer was (SEQ_V4_F; AATGATACGGCGACCACCGAGATCTACACGCCTCCCTCGCGCCATCAGAGATGTG); and the reverse primer was (INDEX_R_bc1 to bc96; CAAGCAGAAGACGGCATA CGAGATXXXXXXXXGTGACTGGAGTTCAGACGTGGCTC), and the amplification was performed using specific PCR cycling conditions (Supplementary Data).

Next, we ran 5 μL from all reactions on a 1% agarose gel to check the availability and size of amplicons/products (~453 bp). Consequently, PCR 4 products were cleaned up as previously described.

Using 2 μL of the cleaned product and a Quant-iTTM PicoGreen^®^ dsDNA Assay kit (Life Technologies, UK), the PCR products were quantified. Each sample was pooled in equimolar proportions, and no more than 20 μL from every reaction was added to the last pool. The pool was then gel-purified, and the necessary size band (approximately 453 bp) was extracted using a QIAEX II kit (Qiagen, UK), following the manufacturer’s recommendations, while eliminating as much agarose gel as feasible. The final pool of samples was measured in triplicate using the Quant-iTTM PicoGreen^®^ dsDNA kit. Specimens were frozen at −80 degrees Celsius until sequencing.

### 2.4. Sequencing on the Illumina MiSeq

The ends of each read were overlapped to generate full-length reads of the V4 region with a high-quality single run using the Illumina MiSeq version 3 reagents and the paired 300-bp reads. The MiSeq run output is estimated to be more than 20 million reads and, assuming 96 indexed samples, can generate more than 100,000 reads per sample; this is commonly recognized as being sufficient for 16S rRNA surveys.

### 2.5. Analysis of Illumina MiSeq Data and Molecular Detection

Paired-end Illumina MiSeq sequences were handled by QIIME (Quantitative Insights into Microbial Ecology; v1.9.1) [26] by removing sequences with a length of <200 and >400 nucleotides. Sequences with an average Phred quality score of less than Q30 were also removed. In addition, mismatched 5′ primers and barcode sequences were deleted from the dataset. We deleted possible chimeric sequences from downstream processing using Chimera Slayer [27].

Using the UCLUST algorithm 19, the sequences were put into representative operational taxonomic units (OTUs) with a 97% sequence identity. These OTUs were then aligned with full-length 16S rRNA marker gene sequences from the Greengenes reference alignment (v_13.8) using PyNAST 20, and their taxonomic identities were determined using the Ribosomal Database Project Classification Tool (V_2.2) with open reference OTU picking, as implemented in QIIME [28]. Several species were discovered in the background of the negative control, but none of them dominated the others to a significant degree. In addition, contaminating sequences were filtered out prior to analysis. The collected dataset was then transformed into a final quality-filtered OTU table, which displayed either absolute normalized counts or relative abundance.

### 2.6. Statistical Analysis

Data were analyzed using SPSS Statistics version 26 (IBM Corporation, USA). The Shapiro–Wilk test for normality was applied. The probability value of *p* < 0.05 was considered statistically significant.

The Shannon–Wiener index, which considers both taxonomic richness and evenness, was used to measure the diversity of the bacterial population in the sputum specimens. These indices were used to evaluate the association between the microbiota structure and the clinical status.

## 3. Results

### 3.1. Participants and Sputum Samples

Eight participants with ACO were included in a longitudinal study with sputum samples obtained in both the EX and ST state. The clinical characteristics of the study participants are included in Table 1. The mean (±SD) participant age was 56.12 years (±13.06); the majority were female (75%) and overweight, with an education level below a Bachelor’s degree. All of the patients received two COVID-19 vaccines, had a smoking history, and had an asthma diagnosis before the age of 40 years. Furthermore, all of the patients had regular typical respiratory symptoms, with an FEV_1_/FVC ratio of less than 0.70 (Table 1).

### 3.2. The Association between Clinical Status and the Respiratory Microbiome in ACO

We investigated the bacterial community in sputum samples from patients in EX and ST states to determine if the clinical condition of ACO patients is connected to the lung microbiome.

**Figure 2:**
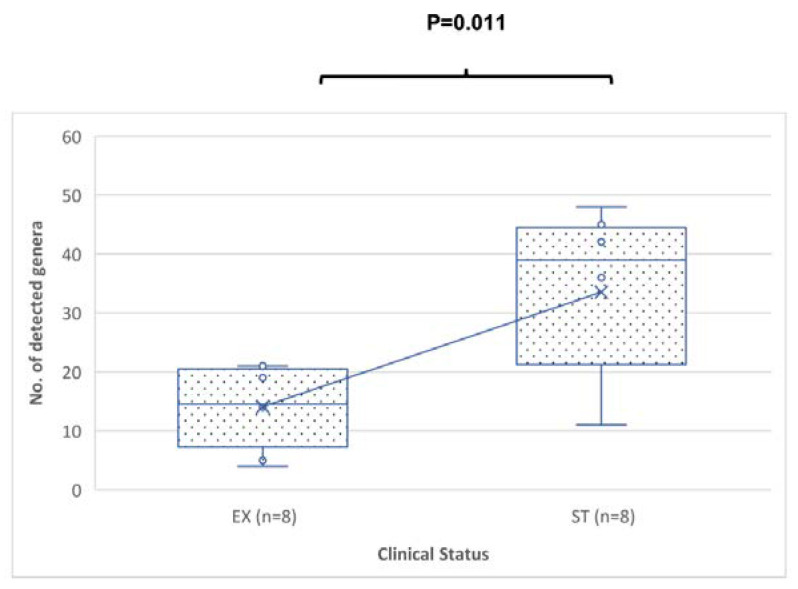
Comparison of the number of genera from sputum specimens from patients in the EX versus ST states. The *p*-value was = 0.011 using the Mann–Whitney U test. The median is represented by a horizontal line inside each box, (x) represents the mean, the interquartile range is indicated by the boxes, and the whiskers denote the range. The numbers of detected genera represent the numbers of any genera with a relative percentage abundance of greater than 0%.

The quantity of discovered genera was substantially more in the ST sputum samples than in the EX sputum samples (*p*-value = 0.011) (Figure 2). The total number of reported genera was the total number of genera having a relative percentage abundance >0%.

The predominant detected genera in the ST sputum samples collected from the ACO patients were *Streptococcus* (38.5%), *Pseudomonas* (13.3%), and *Veillonella* (9.2%). *Streptococcus* (24.1%), *Veillonella* (17.7%), and *Rothia* (11.4%) were predominant in the EX samples (Table 2).

**Figure 3:**
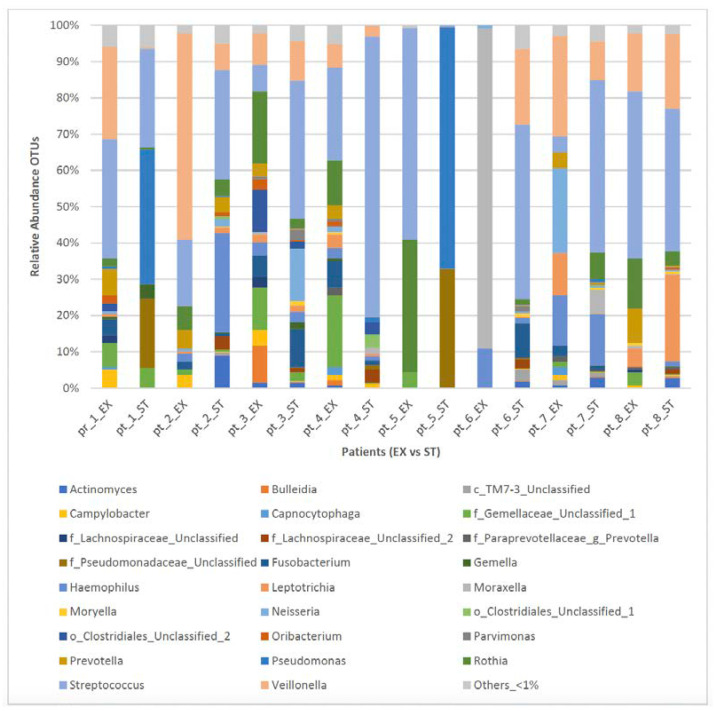
Bacterial taxa distribution (genera-level) of sputum samples from eight ACO patients in the EX versus ST states. Taxa name with “unclassified” suffix might not be assigned to the level of genus and are shown at the lowest known taxon. ST: stable; EX: exacerbation.

In the ST state, the following genera were detected in most of the patients (≥5/8 patients): Veillonella, others_ < 1%, Streptococcus, Haemophillus, Actinomyces, and Rothia. In comparison, in the EX states, Veillonella, others_ < 1%, Streptococcus, Prevotella, Campylobacter, Fusobacterium, Haemophillus, and Rothia were detected in most of the patients (≥5/8 patients) (Table 3). Figure 3 shows the bacterial genera distribution of sputum samples from the eight ACO paired patients in the EX and ST states. As is shown in Table 4, the changes in the lung microbiome were not uniform between the ST and EX states in the ACO patients, and a number of patients showed a high change in the relative abundance of the main genera.

**Figure 4:**
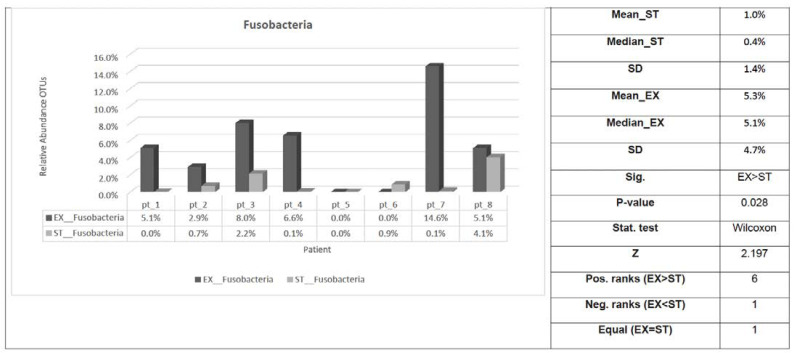
Fusobacteria phylum distribution of sputum samples from eight ACO patients in the EX versus ST states. Sig: indicates whether a statistically significant difference exists (*p*-value < 0.05) between EX and ST; Stat. test: the statistical test used; Z: standardized test statistic; Pos. ranks: the number of positive differences between EX and ST in the paired samples; Neg. ranks: the number of negative differences between EX and ST in the paired samples; Equal: the number of patients where there was no difference between EX and ST. ST: stable; EX: exacerbation; SD: standard deviation.

Only the relative abundance of Fusobacteria phylum was substantially higher in the EX compared with the ST samples (Table 5 and Figure 4).

**Figure 5:**
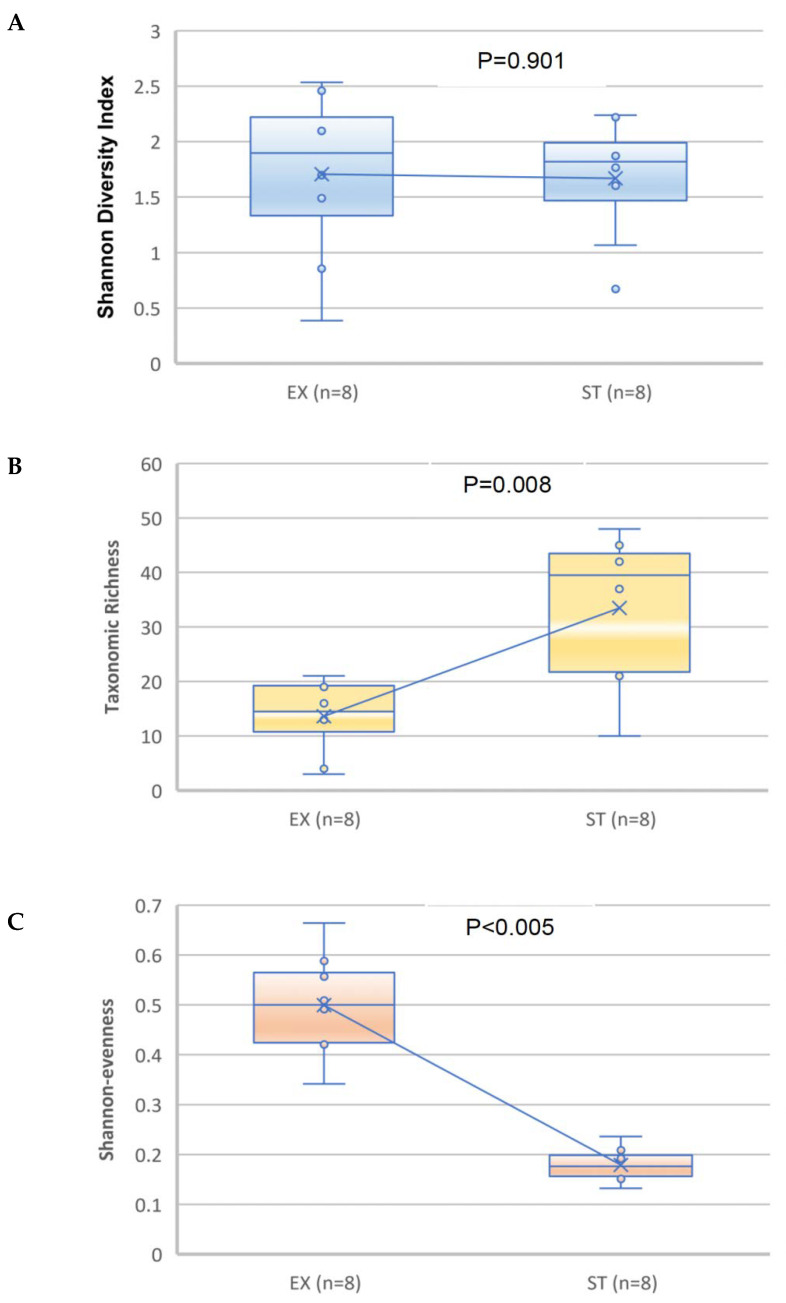
Comparison of the Shannon diversity (**A**), taxonomic richness (**B**), and Shannon evenness (**C**) of the bacterial microbiome for the sputum specimens from patients in the exacerbation versus stable states. The paired Student’s t-test was used. The median is represented by a horizontal line inside each box, the interquartile range is indicated by the boxes and the range is denoted by the whiskers. The line is connecting the means of the two groups.

No significant difference was observed (*p* = 0.901, paired Student’s t-test.) in the diversity of the microbial community (Shannon–Wiener diversity index) (Figure 5A). A significant increase was observed in the median and mean taxonomic richness and a significant reduction was observed in the median and mean evenness of the ST-related sputum samples compared with the EX samples (*p* = 0.008 and <0.005, respectively; paired Student’s t-test) (Figure 5B,C).

## 4. Discussion

The immediate aim of this research was to define the bacterial respiratory microbiome present in patients in a stable state, as well as in the course of an exacerbation of ACO. The NGS Illumina MiSeq was used to accomplish this, comprehensively illuminating the composition of the respiratory microbiome and allowing the detection of rare bacteria associated with culturing difficulties and that, therefore, remain untargeted in the context of typical antimicrobial therapies.

Data that have compared the outcomes of patients with ACO to those of patients with asthma or COPD without overlap are contradictory, most likely as a result of the vast variety of individuals covered under this category. In one population-based cohort followed for a median of nine years, patients with ACO and patients with COPD had comparable risks of exacerbations and all-cause mortality compared to symptomatic smokers without COPD [29]. By contrast, other studies have suggested that patients with ACO may have worse control of their condition in terms of lung function, exacerbation rates, and respiratory symptoms than patients with asthma or COPD who do not have overlap [30,31].

Nonpharmacologic measures for ACO patients are based on practical approaches in asthma and COPD management. Initial strategies for pharmacotherapy outlined by the joint GINA/GOLD statement on ACO are based on expert opinion [4,18]. Usually, the clinical trials of medications for asthma and COPD exclude participants with ACO features. Consequently, large trials that assess clinically essential outcomes in well-defined ACO patients are required. Despite ICS medication, some ACO patients may have exercise limitations or recurrent exacerbations. Virtually no data exist to guide subsequent therapy modifications; a stepwise approach based on symptoms, exacerbations, and response to therapy is feasible [19,20,21].

Molecular methods for characterizing the microbiota of patients with chronic respiratory diseases are innovative. Therefore, the current study, which relied on NGS, provides novel information regarding the sputum microbiota in ACO patients in the EX and ST states. The ACO patients in our study displayed bacterial colonization in the lower airways, even when their condition was characterized as being clinically stable; it is a challenge to conclude the bacterial role in EX. Our investigation found significant differences across the study groups.

The high-throughput Illumina MiSeq utilized in our study uncovered a significantly high bacterial diversity in the sputa. In our study, aerobic bacteria genera such as *Streptococcus, Haemophilus, Pseudomonas,* and *Moraxella* were reported in high abundance, parallel to those reported in previous COPD studies using culture techniques [32,33]. A wide range of taxa was discovered in great abundance, revealing diverse microbial ecosystems in ACO patients’ lungs; *Prevotella, Veillonella*, and *Actinomyces* anaerobes were found at high rates in our study.

Our findings corroborate the data in cystic fibrosis (CF), showing that the microbial populations in the airways can be distributed into two different groups [10,34,35,36]. One category consists of relatively few taxa that dominate the environment. Among our patients with ACO, the aerobic genera dominated the bacterial communities most commonly, including *Streptococcus, Haemophilus, Pseudomonas*, *Moraxella,* and *Rothia*, in addition to the anaerobic genera *Prevotella* and *Veillonella*. The second group encompassed less-encountered taxa with a low abundance, accounting for most of the observed community richness.

It was further observed that patients with ACO in our study had distinctive microbiomes. The composition of the microbial communities in the airways varied from one patient to another. It is worth noting that the patient-specific community structures that showed a dominance of a single or a few genera were consistent with previous studies on COPD patients [10,37,38]. These studies revealed that a distinct profile is retained over time.

Despite the inadequate nature of the literature surrounding the microbiome of the lower airways for ACO patients, studies have confirmed that many of the bacteria present in COPD patients are those that are present in the healthy population [9,10,38,39]. In view of this, it was suggested that a core respiratory microbiome exists, constituting *Streptococcus*, *Pseudomonas*, *Prevotella*, *Fusobacteria*, *Haemophillus*, and *Veillonella*, but the scale of the bacterial community differs depending on whether one is infected or not [10,35].

Most studies in the field of the lung microbiome in patients with COPD have only focused on comparing it to that of healthy subjects or smokers and has been mostly restricted to limited comparisons of phyla levels [10]. However, the debatable issue regarding the role played by bacteria in developing stable COPD is the degree to which the bacterial microbiome of the airways contributes to COPD exacerbations [10,40]. Although bacteria are frequently observed in the context of a COPD exacerbation, the elevated level of bacterial isolation in patients with stable COPD means that the connection between the two is not clear. Accordingly, this research attempted to conclude whether ACO exacerbation is associated with the bacterial microbiome in the lungs, and significant differences were observed between patients in the EX and ST states in our study.

Comparing both groups, there was no significant variation in the Shannon index biodiversity. Taxonomic richness decreased during the EX compared to the ST state (*p* = 0.008), and evenness increased (*p* < 0.005). This suggests that specific bacteria predominate during an exacerbation. According to earlier studies, the diversity of the lung microbiota has been found to be greater [41], decreased [38], or equivalent [37] in patients with COPD, compared with healthy controls. These inconsistent results show that a subject’s lung microbiome diversity is likely complex and not a straightforward function of the disease condition. The discrepancies in the findings may be attributable to the methodology utilized, the severity of the condition of the patients involved, or their demographics. Accordingly, we can hypothesize that variations in the bacterial community of the airways, the emergence of novel species, or the transfer of infection by one species to other lung locations can trigger ACO aggravation.

We observed that the *Prevotella* genus was significantly more abundant in the EX samples than in the ST samples. Anaerobes, including the *Prevotella* and *Veillonella* genera, have been increasingly studied in previous years, and the presence of these bacteria in COPD has been verified using culture-based techniques [42]. It is essential to understand that upper airway contamination may limit the findings [43]. Clinical trials are needed to determine the efficacy of treating these infections. The clinical significance of these pathogens is still unknown. In a previous study, anaerobic *Prevotella* was highly detected in CF patients [44]. In fact, it was considered to be a core member in CF patients [34]. This suggests that some *Prevotella* species may have the potential to contribute to the pathogenicity of lung disease in ACO patients; we observed in our study that the *Prevotella* genus was significantly more abundant in the EX-related samples compared with the ST-related samples. However, we could not determine whether there was any association between the presence of individual bacterial genera and measures of lung function or systemic inflammation.

Identifying the frequency of occurrence of certain bacteria in the lower respiratory tract is not a straightforward matter, which stems primarily from the difficulties associated with acquiring samples that are not contaminated by upper airway secretions [45,46]. One way to avoid this is to use bronchoscopy, as it is notable that investigation depending on sputum for the detection of bacterial infection reflects greater levels of bacterial isolation (bacteria have been identified in more than 50% of stable COPD patients) when compared with bronchoscopy studies [45,47,48]. Although the possibility of contamination of sputum from sources external to the lung (for example, the sinuses, pharynx, and mouth) is a serious one, it should be recognized that the lung microbiome resembles the oropharynx rather than the nose [49,50]. In this study, we followed the protocol that ensures low contamination with upper respiratory and mouth bacteria.

Caution must be applied in interpreting our finding relating to the considerably higher relative abundance of *Pseudomonas* in the ST samples compared with the EX samples. With our study’s relatively small sample size, the findings might not reflect the entire population of ACO patients. Additional longitudinal samples would be needed to assess whether *Pseudomonas* was eradicated in these patients prior to their exacerbation episodes or if it was transiently decreased. However, a study by Millares and co-workers found that, during a COPD exacerbation, the sputum microbiome from patients with *Pseudomonas aeruginosa* colonization was more similar to the sputum microbiome of those without colonization; it was also comparable in terms of similarity to stable colonized COPD patients [51]. Furthermore, it was reported that the predominance of *Pseudomonas*, along with reduced microbiome diversity, was a feature of those with moderate-to-severe COPD (and not mild COPD) [38].

The challenge with NGS is that it detects bacteria of unknown clinical significance. However, given the two samples obtained for each patients (whilst they were in exacerbation and stable states), we supposed the clinical relevance would increase.

Although one of the primary aims of this study was to compare the airway bacterial microbiomes between patients in the ST and EX states as a longitudinal design, the sample size was relatively small. As such, the statistical comparison results may not represent a larger population. However, given the very little research on ACO and its low prevalence, this study should add valuable information and have many advantages over previous studies in this field. Accordingly, our study can be considered a pilot study, and it is recommended that the findings of this study be confirmed using a larger number of ACO patients. 

This study examined the composition of the respiratory microbiome in clinically stable ACO patients and those experiencing an exacerbation. A better knowledge of this link should allow for a more focused administration of antibiotics active against the elevated number and activity of pathogenic bacteria, particularly during exacerbations, hence improving clinical efficacy and patient outcomes.

## 5. Conclusions

This is the first report regarding the respiratory microbiome among ACO patients. Our results suggest that the NGS Illumina Miseq provides a comprehensive analysis of the lung microbiome and enables the detection of less abundant and potential pathogens that are difficult to culture and, as such, are not targeted by conventional antimicrobial therapies.

During an ACO exacerbation, bacterial taxonomic richness decreased and microbiota evenness increased relative to the stable state in ACO patients. This alteration in the airway bacterial community suggests that important pathogens may be replacing much of the airway microbiome during an exacerbation, reducing the microbial richness.

The observation that the *Prevotella* genus was significantly more abundant in the exacerbation state samples compared with the stable state samples may suggest that some *Prevotella* species may have the potential to contribute to the pathogenicity of ACO exacerbations.

Both host and pathogen variables influence the bacterial microbiome. Additional research is necessary to determine the factors that influence the ACO bacterial microbiome.

Disease progression, exacerbations, and treatments, including antibiotics and ICS, can alter the presence of and type of bacteria present in the airways of patients with stable ACO. Bacteria can impact the progression and exacerbations of ACO illness. Antibiotic intervention studies may be required if the association between bacteria and poor outcomes in stable ACO patients can be established.

## Figures and Tables

**Table 1:** Demographics and clinical data for the study participants (N = 8).

Variables	N (%), M (±SD), or Range
**Age**	56.12 (±13.06)
**Gender**	
Male	2 (25.0%)
Female	6 (75.0%)
**Body mass index (BMI)**	29.77 (±4.20)
**Education**	
Below Bachelor’s degree	5 (62.5%)
Bachelor’s degree or higher	3 (37.5%)
**Smoking**	
Current smoker	0 (0.0%)
Previous smoking history	8 (100.0%)
**COVID-19 vaccination status**	
Two doses	8 (100.0%)
**Family history of lung disease (asthma, COPD)**	5 (62.5%)
**Family history of allergic rhinitis**	5 (62.5%)
**Family history of eczema**	1 (12.5%)
**Documented history of asthma before age 40 years**	8 (100.0%)
**No. of exacerbations during the last year**	Median = 3 (2 to 5)
**Typical respiratory symptoms**	8 (100.0%)
**FEV_1_/FVC < 0.70**	8 (100.0%)
**Peripheral blood eosinophil count (>300 cells/microL)**	5/7 (71.4%)
**Post-bronchodilator increase in FEV_1_, range**	7.1 to 11.8%
**Comorbidities**	
Diabetes mellitus	5 (62.5%)
Allergic rhinitis	2 (25.0%)
Cardiovascular disease	7 (87.5%)
**SABA as needed for symptoms**	7 (87.5%)
**Maintenance medication**	
LAMA	1 (12.5%)
ICS/LABA	2 (25.0%)
ICS/LABA/LAMA	5 (62.5%)
**Antibiotic during the exacerbation**	8 (100.0%)

**Abbreviations:** Inhaled corticosteroids (ICS), inhaled corticosteroid/long-acting beta-agonist (ICS/LABA), short-acting beta-agonist (SABA), long-acting muscarinic antagonist (LAMA), oral corticosteroid (OCS).

**Table 2:** The relative percentage of genera detected in the study samples.

Sputum Samples during EX States (n = 8)	Sputum Samples during ST State (n = 8)
*Streptococcus*, 24.1%	*Streptococcus*, 38.5%
*Veillonella*, 17.7%	*Pseudomonas*, 13.3%
*Rothia* 11.4%	*Veillonella*, 9.2%
*Moraxella*, 11.2%	other_ < 1%, 7.1%
Others_ < 1%, 6.6%	f_*Pseudomonadaceae*_Unclassified, 6.7%
f_Gemellaceae_Unclassified_1, 6.1%	*Haemophilus*, 6.1%
*Haemophillus*, 4.2%	*Leptotrichia*, 3.5%
*Prevotella*, 4.2%	*Fusobacterium*, 2.9%
*Neisseria*, 3.4%	*Rothia*, 2.6%
*Leptotrichia*, 3.0%	*Actinomyces*, 2.2%
*Fusobacterium*, 2.8%	*Neisseria*, 2.2%
*Campylobacter*, 2.1%	f_*Lachnospiraceae*_Unclassified_2, 1.6%
o_Clostridiales_Unclassified_2, 1.7%	f_*Gemellaceae*_Unclassified_1, 1.1%
*Bulleidia*, 1.5%	*Moraxella*, 1.1%

The genera shown are in the descending order of the average relative abundance. Taxa name with “unclassified” suffix might not be assigned to the level of genus and are shown at the lowest known taxon.

**Table 3:** The difference between the EX and ST states in terms of the relative abundance of genera detected in the study samples.

	Genera	Pos. Ranks (EX > ST)	Neg. Ranks (EX < ST)	Equal (EX = ST)	Stat. Test	*p*-Value	Sig.
1.	*Actinomyces*	1/8	6/8	1/8	Sign	0.125	**-**
2.	*Bulleidia*	2/8	4/8	2/8	Sign	0.688	**-**
3.	*Campylobacter*	6/8	1/8	1/8	Wilcoxon	0.028	**EX > ST**
4.	*f_Gemellaceae_Unclassified_*1	7/8	1/8	0/8	Wilcoxon	0.017	**EX > ST**
5.	*f_Lachnospiraceae_Unclassified_*2	0/8	6/8	2/8	Wilcoxon	0.028	**ST > EX**
6.	*f_Pseudomonadaceae_Unclassified*	0/8	5/8	3/8	Wilcoxon	0.043	**ST > EX**
7.	*Fusobacterium*	4/8	3/8	1/8	Wilcoxon	1.000	**-**
8.	*Haemophillus*	3/8	4/8	1/8	Sign	1.000	**-**
9.	*Leptotrichia*	4/8	3/8	1/8	Wilcoxon	0.735	**-**
10.	*Moraxella*	3/8	3/8	2/8	Sign	1.000	**-**
11.	*Neisseria*	4/8	4/8	0/8	Wilcoxon	0.779	**-**
12.	*o_Clostridiales_Unclassified_*2	2/8	1/8	5/8	Sign	1.000	**-**
13.	*other_* < 1%	6/8	2/8	0/8	Wilcoxon	0.263	**-**
14.	*Prevotella*	6/8	1/8	1/8	Wilcoxon	0.028	**EX > ST**
15.	*Pseudomonas*	0/8	7/8	1/8	Sign	0.016	**ST > EX**
16.	*Rothia*	6/8	2/8	0/8	Sign	0.289	**-**
17.	*Streptococcus*	3/8	5/8	0/8	Wilcoxon	0.263	**-**
18.	*Veillonella*	4/8	3/8	1/8	Wilcoxon	0.310	**-**

The threshold of 1% relative percentage abundance of genera was used to determine the presence of each genus. Taxa name with “unclassified” suffix might not be assigned to the level of genus and are shown at the lowest known taxon. ST: stable; EX: exacerbation.

**Table 4:** The presence or absence of each genus in the EX and ST states in the study.

	Genera	No. of Patients +ve at EX	No. of Patients +ve at ST	No. of Patients +ve Both at EX and ST
1.	*Actinomyces*	1/8	5/8	1/8
2.	*Bulleidia*	2/8	0/8	0/8
3.	*Campylobacter*	5/8	1/8	1/8
4.	*f_Gemellaceae_Unclassified_*1	7/8	2/8	2/8
5.	*f_Lachnospiraceae_Unclassified_*2	0/8	5/8	0/8
6.	*f_Pseudomonadaceae_Unclassified*	0/8	5/8	0/8
7.	*Fusobacterium*	5/8	4/8	3/8
8.	*Haemophillus*	5/8	6/8	5/8
9.	*Leptotrichia*	4/8	3/8	2/8
10.	*Moraxella*	1/8	2/8	0/8
11.	*Neisseria*	2/8	2/8	0/8
12.	*o_Clostridiales_Unclassified_*2	2/8	2/8	1/8
13.	*other_* < 1%	7/8	7/8	6/8
14.	*Prevotella*	6/8	1/8	1/8
15.	*Pseudomonas*	0/8	3/8	0/8
16.	*Rothia*	5/8	5/8	3/8
17.	*Streptococcus*	6/8	6/8	5/8
18.	*Veillonella*	7/8	7/8	6/8

The threshold of 1% relative percentage abundance of genera was used to determine the presence of each genus. Taxa name with “unclassified” suffix might not be assigned to the level of genus and are shown at the lowest known taxon. ST: stable; EX: exacerbation.

**Table 5:** The difference between the EX and ST samples in terms of the relative percentage of phyla detected in the study.

**Phyla**	**EX (n = 8)**		**ST (n = 8)**		***p*** **-Value**
	M	SD	M	SD	
Actinobacteria	10.3%	13.1%	11.9%	10.7%	0.674
Bacteroidetes	6.8%	6.2%	1.3%	2.4%	0.063
Firmicutes	53.7%	25.9%	68.5%	31.4%	0.401
Fusobacteria	5.3%	4.7%	1.0%	1.4%	**0.028** *
Proteobacteria	23.7%	33.5%	17.2%	34.1%	0.484
Spirochaetes	0.0%	0.0%	0.0%	0.0%	0.059
Synergistetes	0.0%	0.0%	0.0%	0.0%	0.180
TM7	0.2%	0.5%	0.1%	0.1%	0.500
Tenericutes	0.0%	0.0%	0.0%	0.0%	1.000

The Wilcoxon signed-rank test was used to generate the *p*-values. * indicates a significant difference. M: mean; SD: standard deviation.

## Data Availability

Not applicable.

## Supplementary Materials

The following supporting information can be downloaded at: https://www.mdpi.com/article/10.3390/jcm12062118/s1.
