# Supplementary material for: Alteration of the Respiratory Microbiome in Hospitalized Patients with Asthma–COPD Overlap during and after an Exacerbation
Source: J Clin Med. 2023 Mar 8;12(6):2118. doi: 10.3390/jcm12062118 (PMC10051973; doi:10.3390/jcm12062118)
Supplement: Supplementary file 1 [file jcm-12-02118-s001.zip › jcm-2249783-supplementary.pdf]

Supplementary Data

Table S1. Primers used for amplification of the bacterial 16S rRNA gene.

Primer name	Primer sequence (5' - 3')
515F (forward)	GTGCCAGCMGCCGCGGTAA
806R (reverse)	GGACTACHVGGGTWTCTAAT

Table S2. Master mix preparation for the PCR 1 step.

Reagent	Volume
5x Phusion Hifi Buffer	5µl
dNTP (10 mM)	0.5µl
V4 primer mix (10µM)	1µl
Phusion HS II polymerase	0.25µl
DEPC (molecular grade) water	Up to 25µl per reaction

Table S3. PCR cycling conditions for the pre-amplification (PCR 1) step.

Cycle step	Temperature (ºC)	Time	No. of cycles
Initial denaturation	98ºC	30s	1
Denaturation	98ºC	10s	
Annealing	52ºC	30s	10
Extension	72ºC	20s	
Final extension	72ºC	5min	1
Hold at 4°C			

Table S4. Clean-up process of PCR products for PCR 1 to PCR 4 steps.

	**Step A: DNA binding to magnetic beads**
1.	vortex magnetic beads well before use to resuspend any magnetic beads that may have settled.
2.	Aliquot 15-µl of Axygen beads to 10-µl of PCR product into a sterile 96-well plate.<br>**PCR 3 step:** 17.5-µl of Axygen beads was added to 10-µl of the PCR product.<br>**PCR 4 step:** 35-µl of Axygen beads and the entire PCR product was used.
3.	Mix well by repeated pipetting and incubate at room temperature for 5-min.
4.	Place the reaction plate onto the IMAG separation device and wait until the liquid become clear.
5.	Remove the clear liquid from the plate and discard.
	**Step B: Beads washing to remove salt and contaminants**
1.	Add 180-µl of 70% ethanol (EtOH) to each well of the reaction plate and incubate for 30sec at room temperature.
2.	Remove the 70% EtOH from each well and discard (this step was repeated twice).
3.	Air-dry the beads at room temperature for no more than 5-min. over drying the magnetic beads was avoided as over drying will cause the beads to crack and lead to decreased elution efficiency.
4.	We ensured that all the EtOH has been removed from each well.
5.	Add 11-µl of molecular grade water (DEPC H_2O) to each well.<br>**PCR 3 step:** 16-µl of DEPC H_2O.<br>**PCR 4 step:** 50-µl of DEPC H_2O
6.	Remove reaction plate from the IMAG separation device and mix well by gentle vortex to resuspend the magnetic beads.
7.	Place the reaction plate onto the IMAG separation device for 1min to separate the beads from the solution.
	**Step C: Elute purified PCR products from magnetic beads**
	Transfer 10-µl of the cleaned-up PCR product to a sterile 96 well plate for the next PCR step.<br>**PCR 3 step:** 15-µl of the cleaned-up PCR product were transferred.<br>**PCR 4 step:** all of the cleaned-up PCR product were transferred to a sterile 96-well plate.

This table represents the cleaned-up process of PCR products using AxyPrep Mag PCR Clean-up kit (Life Technologies, UK). The steps are common for PCR 1 to PCR 4 steps, unless otherwise specified.

Table S5. Master mix preparation for reverse and forward tagging (PCR 2 and PCR 3) steps.

Reagent	Volume
5x Phusion Hifi Buffer	5µl
dNTP (10 mM)	0.5µl
Reverse_MT_tag Primer mix (0.5µM)	2µl
Phusion HS II polymerase	0.25µl
DEPC water	7.25µl

Table S6. PCR cycling conditions for the PCR 2 and PCR 3 steps.

Cycle step	Temperature	Time	No. of cycles
Initial denaturation	98°C	60s	1
Denaturation	98°C	10s	
Annealing	50°C	30s	1
Extension	72°C	60s	
	hold at 4°C		

Table S7. Master mix preparation for the PCR 4 step.

Reagent	Volume
5x Phusion Hifi Buffer	10µl
dNTP (10 mM)	1µl
forward primer (SEQ_V4_F)	2.5µl
reverse primer (INDEX_R_bc1 to bc96	2.5µl
Phusion HS II polymerase	0.5µl
DEPC water	7.25µl

Table S8. PCR cycling conditions for the PCR 4 step.

Cycle step	Temperature	Time	No. of cycles
Initial denaturation	98°C	30	1
Denaturation	98°C	10	
Annealing	63°C	30	34
Extension	72°C	30	
	hold at 4°C		
